# Unveiling the potential of *Lactobacillus johnsonii* in digestive diseases: a comprehensive review

**DOI:** 10.3389/fmicb.2025.1508382

**Published:** 2025-08-01

**Authors:** Jinjie Zhou, Shuang Ma, Zhuo Huang, Qinyan Yao, Zihan Yu, Jihua Chen, Liuqing Yao, Lanping Zhu, Xin Chen

**Affiliations:** ^1^Department of Gastroenterology and Hepatology, General Hospital, Tianjin Medical University, Tianjin, China; ^2^Tianjin Institute of Digestive Diseases, Tianjin, China; ^3^Tianjin Key Laboratory of Digestive Diseases, Tianjin, China

**Keywords:** *Lactobacillus johnsonii*, digestive diseases, intestinal infections, gut microbiota, inflammatory bowel disease, brain-gut axis

## Abstract

**Background:**

*Lactobacillus johnsonii* (*L. johnsonii*) is a lactic acid-producing probiotic, possessing the potential to modulate intestinal microbiota balance, which can enhance immune function, and reduce the risk of intestinal infections. In recent years, increasing studies have demonstrated the positive impact of this strain and its metabolites on the health of multiple systems, including the stomach, intestine, liver, and brain.

**Objective:**

This article aims to systematically review the mechanisms of action and clinical application progress of *L. johnsonii* in the prevention and treatment of digestive system-related diseases. The focus is on exploring its systemic regulatory role through the “microbiota-gut-organ axis.”

**Methodology:**

By collating and analyzing recent research findings on *L. johnsonii*, we evaluated its specific mechanisms in regulating intestinal barrier function, immune response, and neuroendocrine signaling pathways. This strain can be isolated and identified through experimental means, and its population abundance can be quantitatively analyzed, providing a basis for studying its biological functions.

**Results:**

Studies have revealed that *L. johnsonii* exhibits significant interventional potential in various cross-system diseases, including inflammatory bowel disease (IBD), intestinal infections, *Helicobacter pylori* (*H. pylori*)-associated gastritis, non-alcoholic fatty liver disease, and neurodegenerative diseases. These findings further validate the important role of this strain in maintaining intestinal microbiota homeostasis, regulating body metabolism, and brain-gut axis function.

**Conclusion:**

In summary, *L. johnsonii* shows great promise in digestive health by modulating immunity, enhancing the gut barrier and balancing gut microbiota. Future research should further explore its mechanisms of action, aiming to provide a solid theoretical foundation and experimental support for its precise therapeutic reality.

## 1 Introduction

Digestive diseases are an important public health problem worldwide, affecting millions of people and imposing a huge medical and socio-economic burden. The digestive system, including the oral cavity, esophagus, stomach, intestines and liver, plays a central role in nutrient absorption, immune regulation and maintenance of metabolic homeostasis. In recent years, the incidence of IBD, irritable bowel syndrome, and gastrointestinal infections has continued to rise globally (Molodecky et al., 2012; Peery et al., 2022; Ricciardiello, 2022; Roy and Dhaneshwar, 2023), and there is an urgent need to explore new intervention strategies and therapeutic approaches.

A growing body of evidence suggests that the gut microbiota-a complex ecosystem of diverse microorganisms-plays a critical role in maintaining digestive and systemic health. An imbalance in the composition and function of the microbiota can lead to a variety of diseases including IBD, liver disease, metabolic syndrome and even neuropsychiatric disorders, with mechanisms of action involving immune regulation, epithelial barrier function, and the gut-brain axis (Cryan and Dinan, 2012; Rooks and Garrett, 2016; Fan and Pedersen, 2021). Therefore, strategies to regulate gut microbiota, especially the use of probiotics, have become a hot topic of current research in the prevention and treatment of digestive disorders. Probiotics are defined by the World Health Organization as “active microorganisms that are beneficial to the health of the host when ingested in adequate amounts” (Mu et al., 2018) and have been gradually applied in clinical interventions.

Among many probiotic candidates, *L. johnsonii* has drawn much attention. This is due to its robust colonization capacity, immunomodulatory potential, and diverse mechanisms of action. It was originally classified as *L. acidophilus*. *L. johnsonii* is a Gram-positive, non-bacteriophage-forming, partially anaerobic lactic acid bacterium. It is widely found in the gastrointestinal tract of both humans and animals (Yang et al., 2020). Like other *Lactobacillus* species such as *L. acidophilus*, *L. johnsonii* may contribute to host health in several ways. It can adhere to intestinal epithelial cells. It may enhance the mucosal barrier function. It also produces antimicrobial substances and modulates both innate and adaptive immunity (de Roos and Katan, 2000; Zhang et al., 2021). Specific strains of *L. johnsonii* have shown anti-inflammatory, antipathogenic, and metabolic-modulating properties. These traits suggest potential therapeutic value in conditions such as IBD, intestinal infections, liver diseases, and gut-brain axis disorders. Advances in histological techniques have deepened our understanding of its molecular mechanisms. Researchers have identified key proteins, such as GroEL and GAPDH, which are crucial in bacterium-host interactions (Xie et al., 2019). However, the functional differences and dose-dependent responses among strains suggest that precise screening and validation are still needed in probiotic development. A comprehensive 2024 review highlighted that probiotics-including specific Lactobacillus strains-modulate the gut-brain axis by regulating neuroinflammation, neurotransmitter production, and cognitive function in human trials involving mild cognitive impairment and dementia. This supports the potential of *L. johnsonii* and related probiotics in gut-brain disorders (Fekete et al., 2024).

This review systematically summarizes the current research progress of *L. johnsonii* in a variety of digestive diseases, covering its mechanism of action and potential clinical value in IBD, gastrointestinal infections, gastritis, obesity, hepatopathy, regulation of the gut-brain axis, gut microbiota dysbiosis, and colorectal cancer. We integrated representative results from animal experiments, cellular models and clinical studies (Tables 1–3), focusing on the analysis of its functional properties in regulating the intestinal barrier, metabolic intervention,maintenance of microbiota homeostasis and immune modulation. Currently, there are relatively few studies on the role of the specific strain *L. johnsonii* in digestive system diseases. This article provides readers with a comprehensive and focused update on the specific strain *L. johnsonii*, avoiding generalizations. This review not only describes the role of *L. johnsonii* within the gut but, more importantly, systematically and thoroughly explains how it influences distant organs such as the liver, brain, heart, and lungs through these axises. It systematically covers the research progress of *L. johnsonii* in almost all major digestive system-related diseases. This review also places significant emphasis on the differing effects of various *L. johnsonii* strains in specific disease models or clinical contexts. This review aims to provide theoretical support for the targeted use of *L. johnsonii*. It also lays a foundation for developing future precision probiotic interventions.

**Table 1:** Therapeutic efficacy and potential mechanisms of *Lactobacillus johnsonii* strains in various of the digestive diseases in animal models.

*Lactobacillus johnsonii* strain	Reference	Experimental model/participant	Disease	Effect/Outcome	Mechanism of action
*FHubES1M16, FI9785, BS15, NCC_533, DPC_6026, N6.2, Byun-jo-01, IDCC9203*	Xie et al. (2019)	Fermented yak milk and fecal	Taxonomic identification of *Lactobacillus*	*Lactobacillus* could be distinguished.	The groEL gene is suitable for designing primers to distinguish between *Lactobacillus* species.
*La1(NCC 553)*	Bergonzelli et al. (2006)	Mice Rabbits	Investigate the GroEL protein of a *La1*	*La1* GroEL binds to mucins and intestinal HT29 cells in a pH-dependent manner.	*La1* GroEL protein attaches to mucus and epithelial cells, and mediates the aggregation of *Hp*.
*IDCC9203* and other	Je et al. (2018) and Jia et al. (2022)	Mice mode UC patients	IBD	Uppressed inflammatory lesions and improve the contraction of colon length.	TLR1/2-STAT3 pathway. Downregulation of the expression of proinflammatory cytokines.
*HF 57*	Isani et al. (2018)	NEC rats	NEC	*L. johnsonii* didn’t protected against NEC.	*L. murinus HF12* is associated with low NEC scores.
*L531* and other	Xia et al. (2020), Yang et al. (2020), Zhang et al. (2021), and Yang et al. (2022)	Piglets	*S. infantis* and *C. rodentium* infection	Attenuated *S. infantis* and *C. rodentium*-induced intestinal damage.	Regulat the NOD activation, ER stress and autophagy. Activate NF-κB signaling pathway
Unspecified	Ma et al. (2022)	Mice	Diarrhea and colitis	Alleviates colitis.	PHB promotes biofilm formation enhance sulfomucin secretion and maturation in a *L. johnsonii*-dependent manner.
*La1*	Sgouras et al. (2005)	Mice	*Hp*-associated gastritis	Attenuate *Hp*-induced gastritis in early stage.	Reduction of pro-inflammatory chemotactic signals responsible.
*456*	Davoren et al. (2019)	Mice	Inhibit gut pathogens and adhere to host mucosa.	*L. johnsoni 456* maintained higher viability at lower pH conditions.	Altered pathogen adhesion.
*JNU3402 (NV-LJ3402)* and other	Yang et al. (2020) and Liu et al. (2022)	Mice, rats	Diet-induced obesity	Ameliorate diet-induced obesity and hyperlipidemia.	PPARγ pathway. “Reconfigure” the gut microbiota.
Unspecified	Chuang et al. (2016)	Rats	Liver injury induced by alcohol	Hepatoprotective properties induced by alcohol.	Reduced AST, ALT, γ-GT and malondialdehyde (MDA) levels and inhibit MDA production.
*BS15*	Xin et al. (2014) and Zhang et al. (2021)	Mice, rats	NAFLD	Effective in preventing NAFLD. Improve intestinal mucosal barrier permeability.	Positive effect on liver lipid peroxidation; inhibited the insulin resistance; attenuated mitochondria abnormalities.
*BFE6154*	Yoon et al. (2023)	Mice	Diet-induced hypercholesterolemia	Regulate cholesterol metabolism.	Reduced TC and LDL and HDL in the blood.
*BS15* and other	Jang et al. (2018), Sun et al. (2020), Wang et al. (2020, 2021), and Sun et al. (2022)	Mice	Memory dysfunction	Modulating the gut-brain axis to ameliorate alcohol-mediated, fluorine induced.	Reduced alcohol unconsciousness time, blood alcohol concentration, and serum AST and ALT levels. Modulated the hypothalamic–pituitary–adrenal axis.
Unspecified	Ekmekciu et al. (2017)	Mice	Restore immune functions	Restore individual functions of intestinal and systemic immunity.	Increase CD4 + and CD8 + cell numbers in the small intestine and spleen and maintained colonic IL-10 production.
Unspecified	Bereswill et al. (2017)	Mice	*C. jejuni* infection	Alleviate intestinal, extra-intestinal and systemic secretion of pro-inflammatory mediators.	Ameliorate colonic apoptosis and impacted adaptive immune cell.
Unspecified	Charlet et al. (2020)	Mice	Colitis mediated by TLR9	Reduce the overgrowth of bacterium populations and reduce inflammatory parameters.	Induced a degradation of the fungal cell wall, decreased pro-inflammatory mediators and enhanced the antiinflammatory cytokine response.
*ATCC11506; ATCC, Manassas, VA, USA*	Fan et al. (2023)	Mice	SILI	Treat SILI.	*L. johnsonii*-dependent immune regulation and IL-10(+) M2 macrophage production.
*ETEC K88*	Tao et al. (2025)	Piglets	Diarrheic	Ameliorate diarrheic disease symptoms.	LJ-EVs modulate macrophage phenotypes.
*GLJ001*	Cai et al. (2025)	Mice	IBD	Prevented DSS-induced colitis.	Gut microbiota-SCFAs axis.
*BNCC 135265*	Chen et al. (2025)	Mice	IBD	Preventive effect on IBD.	Protects intestinal barrier integrity, increases the concentrations of butyric and propionic acids; MAPK signaling pathway.
Unspecified	Makusheva et al. (2024)	Mice	IBD	Recovery efficacy of *L. johnsonii* and *E. faecalis*.	Decrease in the amount of *E. faecalis*. Eliminated protozoan overgrowth.
Unspecified	Li et al. (2024)	Mice	IBD	Treat colitis.	Activate the Nrf2/HO-1 antioxidant signaling pathway.
heat-killed *L. johnsonii*	Zhang et al. (2024)	Mice	IBD	Treat colitis.	*L. johnsonii*-ILA-AhR axis.
heat-killed *L. johnsonii*	Yin et al. (2025)	Mice	Alcohol-associated liver disease	Treat alcohol-associated liver disease.	NOD2-IL-23-IL-22 innate immune axis, help correct the gut microbiota dysbiosis.

*L. johnsonii*, *Lactobacillus johnsonii*. UC, ulcerative colitis. IBD, Inflammatory bowel disease. NEC, Necrotizing enterocolitis. *L. murinus*, *Lactobacillus murinus*. *S. infantis*, *Salmonella Infantis*. *C. rodentium*, *Citrobacter rodentium*. ER, endoplasmic reticulum. PHB, Poly-β-hydroxybutyrate. Hp, Helicobacter pylori. PPARγ, peroxisome-activated receptor-γ. AST, aspartate aminotransferase. ALT, alanine aminotransferase. γ-GT, gamma-glutamyl transferase. MDA, malondialdehyde. NAFLD, Non-alcoholic fatty liver disease. TC, total cholesterol. LDL, lolow-density lipoprotein. HDL, high-density lipoprotein. IL, interleukin. *C. jejuni*, *Campylobacter jejuni*. TLR, Toll-like receptors. SILI, Sepsis-induced liver injury. LJ-EVs, *L. johnsonii* derived extracellular vesicles. SCFA, short-chain fatty acids. MAPK, Mitogen-Activated Protein Kinase. *E. faecalis*, *Enterococcus faecalis*. ILA, indole-3-lactic acid. AhR, aryl hydrocarbon receptor. NOD2, Nucleotide-binding oligomerization domain 2.

**Table 2:** Therapeutic efficacy and potential mechanisms of *Lactobacillus johnsonii* strains in various of the digestive diseases in cell models.

*Lactobacillus johnsonii* strain	Reference	Experimental model/participant	Disease	Effect/Outcome	Mechanism of action
*La1*	Neeser et al. (2000)	Caco-2	Carbohydrate-binding specificities of *La1 in vitro*.	Enable to adhere to the host gut mucosa.	An Endo-H treated yeast cell wall mannoprotein carrying mainly Olinked oligomannosides; the gangliotri- and gangliotetra-osylceramides (asialo-GM1).
*MG*	Bai et al. (2022)	Caco-2	Damaged by H_2_O_2_	The tight junctions of Caco-2 cells damaged by H_2_O_2_ were repaired by incubation with *L. johnsonii MG*.	Promotes the barrier function integrity in Caco-2 cells via GAPDH-JAM-2 binding.
*La1*	Vidal et al. (2002)	IECs	Neonatal immunity and inflammation	Prevent an exaggerated inflammatory response.	Neither *La1* nor its LTAs stimulated IECs.
*L531*	Chen et al. (2021)	IPEC-J2 cells	*S. typhimurium* Infection	Alleviates the Damage Caused by *S. typhimurium*.	Inhibit the TLR4/NF-κB/NLRP3 inflammasome signaling pathway.
*La1*	Granato et al. (2004)	Caco-2 and HT29	mucin-binding capacity	Binds to the intestinal mucosa and participate in gut homeostasis.	EF-Tu has an important role in La1 mucin binding capacity.

Johnsonii, *L. Johnsonii*. IECs, intestinal epithelial cells. LTAs, lipoteichoic acids. *S.* *Typhimurium*, *Salmonella* Typhimurium. EF-Tu, elongation factor Tu.

**Table 3:** Therapeutic efficacy and potential mechanisms of *L. johnsonii* strains in various diseases of the digestive system in humans.

*Lactobacillus johnsonii* strain	Reference	Experimental model/participant	Disease	Effect/Outcome	Possible Mechanism of action.	*p* value
*IDCC 9203*	Shin et al. (2021)	Participants		A higher relief of abdominal pain and bloating.	Unspecified.	<0.05
*La1*	Gianotti et al. (2010)	Patients	CRC	Affect intestinal microbiota.	Modulate DC and T-cell specific responses via the release of anti-inflammatory cytokines.	<0.05
*La1*	Gotteland et al. (2008)	Children	Asymptomatic *Hp* colonization	Be useful in the management of asymptomatic children colonized by *Hp*.	Unspecified.	<0.01
*La1* (NCC533)	Fukushima et al. (2007)	The elderly	As enterally fed	Suppressing infections.	Nutritional status improvement, immune enhancement and anti-inflammation.	<0.05
*La1*	Brunser et al. (2006)	Infant	Breast feeding	The presence of higher counts of bifidobacteria and lactobacilli in the microbiota.	Unspecified.	<0.001
*La1*	Yamano et al. (2006)	Women	Improvement of gut microbiota	Increase total *Bifidobacterium* and *Lactobacillu*s, and decrease lecithinase-positive *Clostridium* in the feces.	The lipoteichoic acid of *La1* and asialo-GM1 of Caco-2 cells are involved in the mediation of adhesion.	<0.05
Unspecified	Pantoflickova et al. (2003)	Volunteers	*Hp*-related gastritis	Have wake favourable effect on *Hp* associated gastritis, particularly in the antrum.	Increase mucin expression and secrete antibacterial substances.	<0.05
*La1*	Cruchet et al. (2003)	Children	*Hp* colonization	Modulate *Hp* colonization in children infected.	Unspecified.	0.0074
*La1*	Felley et al. (2001)	Volunteers	*Hp*-related gastritis	*Hp* infection and gastritis can be down-regulated.	Interfere with enteropathogenic bacteria, secrete anti-bacterial substances and modify the immunological response of the host.	<0.05

*L. johnsonii*, *Lactobacillus johnsonii*. CRC, colorectal cancer. DC, dendritic cell. Hp, *Helicobacter pylori*.

It is important to emphasize that the effects of probiotics are highly strain-specific. Even among different strains of *L. johnsonii*, there may be significant differences in their physiological properties, metabolites, host interaction mechanisms, and ultimate health effects. The results of the studies cited in this review are based on strain-specific experimental models or clinical observations unless otherwise stated, and the results cannot be simply extrapolated to other *L. johnsonii* strains or the species as a whole.

## 2 Infection-associated intestinal diseases

### 2.1 The infection of *Salmonella*

Infection-associated intestinal diseases caused by *Salmonella* lead to inflammation-induced dysbiosis (Gresse et al., 2017; Gillis et al., 2018). Microbiota-derived short-chain fatty acids (SCFAs) promote the growth of regulatory T cells and have anti-inflammatory effects (Atarashi et al., 2013). Microbiota-derived butyrate works with regulatory T (Treg) cells to activate epithelial cells via peroxisome-activated receptor-*γ* (PPARγ) signaling (Byndloss et al., 2017). This activation impacts colonocyte metabolism, which is essential for restricting oxygen availability and maintaining the dominance of anaerobic bacteria. In newly weaned piglets, *Salmonella infantis* infection has a detrimental effect on gut microbiota diversity, leading to enteritis, diarrhea, fever, and stunted growth (Byndloss et al., 2017; Zhang et al., 2017). Salmonella triggers intestinal inflammation, causing an increase in nitrate and oxygen concentrations. This, in turn, depletes SCFAs and creates a favorable environment for *Salmonella* growth, ultimately resulting in severe diarrhea (Drumo et al., 2015; Byndloss et al., 2017). It has been proven that *L. johnsonii N5* and *N7* have antagonistic effects against *Salmonella* with a high survival rate in acidic and bile environments (Wang et al., 2024).

**Figure 1:**
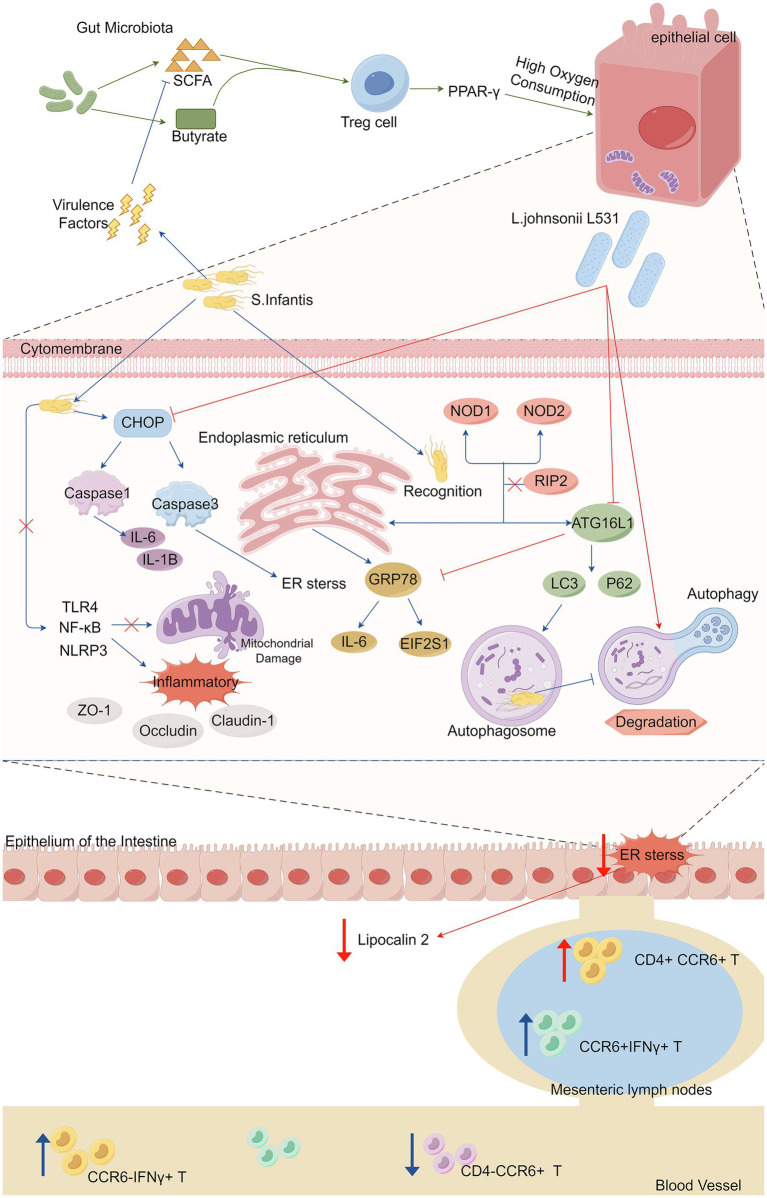
Microbiota-derived short-chain fatty acids (SCFAs) promote the maturation and expansion of Treg cells in the gut and exert profound anti-inflammatory effects, activating intestinal epithelial cells through PPAR-*γ* signaling to drive high oxygen-consuming metabolism. *Salmonella* use their virulence factors to trigger intestinal inflammation by depleting the action of commensal bacteria that produce (SCFAs), subsequently reducing the concentration of SCFAs and promoting Salmonella growth. *Salmonella infantis* infection activated both the NOD pathway and ER stress *in vivo* and *in vitro*, and *Salmonella typhimurium* augmented the inflammatory response by activating the TLR 4/NF-κB/NLRP 3 inflammasome signaling pathway (activation of which has also been linked to the inhibition of mitochondrial damage), which in turn reduced the expression levels of the tight junction proteins ZO-1, Occludin and Claudin-1. *Salmonella infantis* infection significantly increased the expression of ATG16L1 and IRGM in enterocytes, induced the accumulation of LC3 protein, which promotes autophagy, and utilized autophagy and ER stress to promote their immune escape and multiplication. CHOP is the most important pro-apoptotic transcription factor during endoplasmic reticulum stress because it activates caspase 3. CHOP is a key mediator of the maturation process of IL-1B via key mediator of caspase 1 activation, thereby inducing cell death in a mitochondria-dependent pathway. *L. johnsonii* modulates IRE 1A/CHOP and ATF 6A/CHOP signaling to abrogate ER stress-associated apoptosis, promotes ER homeostasis and autophagosome degradation, and inhibits overactivation of associated inflammatory signaling pathways, reduces overexpression of inflammatory cytokines, and contributes to the maintenance of tight junction integrity. In mesenteric lymph nodes, the proportion of CCR 6^+^ IFNγ^+^ T cells was elevated after stimulation by *Salmonella infantis*, and *L. johnsonii L531* induced an increase in the proportion of CD 4^+^CCR 6^+^ T cells. *Salmonella infantis* attack decreased the percentage of CD 4^−^ CCR 6 ^+^ T cells but increased the percentage of CCR 6^−^ IFNγ^+^ T cells in peripheral blood. *L531*, *Lactobacillus johnsonii L531*. SCFA, short-chain fatty acids. Treg cell, regulatory T cell. *S, Infantis. Salmonella Infantis*. IL-6, Interleukin-6. IL-1B, interleukin-1B. ER stress, endoplasmic reticulum stress. CCR 6, CC-chemokine ligand.

Pretreatment of piglets with *L. johnsonii L531* reduces *Salmonella*-induced diarrhea and limit the overgrowth of intestinal bacteria. As aerotolerant anaerobes, *Lactobacillus* species, including *L. johnsonii L531*, can tolerate oxygen briefly and modify gut microbiota composition and SCFA production (Derrien and van Hylckama Vlieg, 2015). Oral administration of *L. johnsonii L531* to newly weaned piglets decreases *Salmonella* colonization in colonic and jejunal contents and accelerates *Salmonella* clearance in feces after infection. It also ameliorates SCFA depletion caused by *S. infantis* infection (He et al., 2019). *L. johnsonii L531* inhibits ER stress and the production of lipocalin 2 in intestinal mucosa, while promoting CCR6 + T-cell responses. Pretreatment increases the proportion of CD4 + CCR6 + T cells in mesenteric lymph nodes, protecting against intestinal inflammation (Zhang et al., 2017; Yang et al., 2020). A research team found that *L. johnsonii L531* ameliorates *S. infantis*-associated enteritis by regulating inflammasomes and NF-κB-SQSTM1 mitophagy signaling pathway and suppressing mitochondrial damage (Xia et al., 2020). It also attenuates intestinal structural and cellular ultrastructural damage. *L. johnsonii L531* protects against *S. infantis*-induced intestinal damage by regulating NOD activation, ER stress, and autophagy (Yang et al., 2022), while NOD1 and NOD2 activate NF-κB through RIP2, promoting intestinal inflammation (Keestra et al., 2011; Xia et al., 2020; Chen et al., 2021), or inhibiting ER stress correlated with ATG16L1 and IRGM expression (Huang and Brumell, 2014; García-Gil et al., 2018; Yang et al., 2022). The specific mechanism is shown in Figure 1.

In conclusion, *L. johnsonii L531* effectively protects against *Salmonella*-induced intestinal inflammation by restoring microbiota balance, preserving SCFA levels, and modulating immune responses. Its regulatory role in ER stress, mitophagy, and NOD signaling highlights its therapeutic potential. These findings support *L. johnsonii* as a promising candidate for preventing infection-associated enteritis.

### 2.2 The other inflammation of intestine

*Lactobacillus johnsonii* has demonstrated the ability to inhibit intestinal inflammation instigated by other bacterial infections. For instance, *L. johnsonii L531* effectively curbs robust inflammatory responses and diminishes the overexpression of cytokines, thereby safeguarding the integrity of tight junctions that are challenged by *Salmonella Typhimurium (S. typhimurium)*, as shown in Figure 1 (Yang et al., 2022). *L. johnsonii N5* promotes the expansion of MHCII+ and CD103 + dendritic cell populations within intestinal Peyer’s patches, augments the number of Treg cells, and mitigates colitis (Yuan et al., 2023). Compared to *Escherichia coli (E. coli)*, *L. johnsonii* recolonization leads to higher numbers of CD4 + and CD8 + cells in the small intestine and spleen, indicating its potential to restore immune functions (Ekmekciu et al., 2017). Additionally, *L. johnsonii NJ13* and *L. johnsonii L531* are capable of suppressing the secretion of pro-inflammatory cytokines and inhibiting the activity of the Toll-like receptors 3 (TLR3) inflammasome (Wang et al., 2015; Zou et al., 2020).

The lipoteichoic acids (LTAs) derived from *L. johnsonii La1* impede the soluble CD14 (sCD14)-mediated response of intestinal epithelial cells (IECs) to lipopolysaccharide (LPS) (Vidal et al., 2002). In addition, elongation factor Tu (EF-Tu), a novel *L. johnsonii La1* bonding factor, plays an important role in the mucin-binding capacity of *L. johnsonii La1*. EF-Tu recombinant protein induces a proinflammatory response in the presence of soluble CD14 (Granato et al., 2004). *L. johnsonii La1* also has probiotic properties enhancing gut microbiota (Yamano et al., 2006). Researches have demonstrated *La1* could enhance the nutritional and immunological status of the elderly, suppressing infections (Fukushima et al., 2007), and *La1* might be beneficial in preventing bacterial translocation in cirrhosis (Chiva et al., 2002), mainly attributed to the antioxidant properties of vitamin C and glutamate (Soriano et al., 2012). Cloned and expressed Bile-Salt-Hydrolase (BSH) genes from *La1* exhibited anti-*Giardia* activities (Allain et al., 2017). Another finding has demonstrated that BaWeiBaiDuSan can promote the growth of *L. johnsonii* and its treatment for sepsis-induced liver injury. The underlying mechanism may involve *L. johnsonii*-mediated immune regulation and the production of interleukin-10 (IL-10)(+) M2 macrophages (Fan et al., 2023).

The regulation of gut microbiota is crucial for combating intestinal bacterial infections and reducing inflammation. Additionally, studies have shown the role of *L. johnsonii 6,084* in reducing sepsis mortality by rebalancing gut microbiota (Han et al., 2022). And the beneficial effects of *L. johnsonii YH1136* in protecting against intestinal dysfunction in high-altitude environments have been revealed (Wan et al., 2022). Prophylactic *L. johnsonii* treatment ameliorates *Campylobacter jejuni* (*C. jejuni*)-induced colonic apoptosis and alleviates pro-inflammatory mediator secretion (Bereswill et al., 2017). *L. johnsonii* protects against intestinal inflammation caused by *Citrobacter rodentium (C. rodentium)* by suppressing immune cell infiltration, cytokine secretion, and ER stress-related apoptosis, and regulates IRE1A/CHOP and ATF6A/CHOP signaling pathways (Bereswill et al., 2017; Zhang et al., 2021). Additionally, *L. johnsonii* induce fungal cell wall degradation, and inhibit *Candida* growth (Charlet et al., 2020). In combination with other probiotics, *L. johnsonii* contributes to enhancing the diversity and composition of the gut microbiota. This results in a reduction in the abundance of harmful bacteria and alleviates diarrhea induced by enterohaemorrhagic *Escherichia coli* (EHEC) (Hu et al., 2021). Moreover, pre-supplementation of *Leuconostoc pseudomesenteroides* and *L. johnsonii* mitigates gut microbiota dysbiosis and diarrhea triggered by enteroinvasive *Escherichia coli* (EIEC) (Wang et al., 2019). *L. johnsonii* derived extracellular vesicles (LJ-EVs) might shut down ERK and NLRP3 activation in intestinal epithelial cells in order to activate M2 macrophages which mitigated the adverse impacts induced by ETEC K88 (Tao et al., 2025). These findings suggest the potential of *L. johnsonii* in regulating gut microbiota homeostasis for intestinal health.

Necrotizing enterocolitis (NEC), a severe intestinal inflammatory disorder in preterm infants, significantly contributes to neonatal morbidity and mortality (Hull et al., 2014; Gupta and Paria, 2016). A study explored the potential of three *Lactobacilli* species, namely *L. murinus*, *L. acidophilus*, and *L. johnsonii*, in relation to NEC. The findings revealed that *L. johnsonii* did not offer protection against NEC (Isani et al., 2018). Another research indicated that the supplementation with *Lactobacillus rhamnosus* and *L. johnsonii* failed to elicit predictable alterations in the fecal microbiota or the overall growth of children afflicted with short bowel syndrome (SBS) (Piper et al., 2020).

*Lactobacillus johnsonii* exhibits strong potential in mitigating intestinal inflammation caused by diverse pathogenic infections through barrier protection, immune modulation and microbiota regulation. However, its variable efficacy in diseases like NEC and SBS highlights the need for more targeted investigations. Future research should focus on elucidating strain-specific mechanisms, understanding host-dependent responses, and characterizing active metabolites such as SCFAs and extracellular vesicles. Integrating multi-omics technologies will help uncover precise host–microbe interactions, while well-designed clinical trials are essential to validate its safety and therapeutic efficacy. A deeper mechanistic understanding will be key to translating the experimental promise of *L. johnsonii* into therapeutic reality.

Considerable variability among strains further complicates the interpretation of *L. johnsonii*’s role in NEC prevention. The article highlights that different *Lactobacillus* species or even strains within the same genus exhibit markedly distinct effects. For example, while *L. murinus HF12* was associated with reduced NEC severity, *L. johnsonii* itself failed to demonstrate comparable protective benefits in similar experimental models. These findings suggest that significant functional differences exist at the strain level, which have not been fully addressed or standardized in clinical practice.

Collectively, these inconsistencies underscore the urgent need for future studies to implement precise strain identification and selection, alongside comprehensive characterization of probiotic properties. Furthermore, clinical trials should incorporate rigorous strain-level distinctions and consider host-specific factors to clarify the true therapeutic potential of *L. johnsonii* in NEC management and to reconcile the observed gap between experimental data and clinical outcomes.

### 2.3 Colorectal cancer

Several studies have examined the correlation between *L. johnsonii* and colorectal cancer (CRC), but findings may be limited. In a particular experimental study, the co-transplantation of *L. johnsonii* and *Bifidobacterium animalis* in ApcMin/+ mice that were concurrently administered 5-fluorouracil (5-FU) resulted in a discernible reduction in polyp burden (Yin et al., 2022). Another study showed that strain *L. johnsonii La1*, not *BB536*, adhered to colonic mucosa, reducing pathogen concentration and modulating local immunity via gut microbiota (Gianotti et al., 2010). The supplementation with either *L. johnsonii* or protocatechuic acid (PCA), a metabolite produced by *L. johnsonii*, effectively arrested the progression of chronic stress-induced CRC. This was accomplished by downregulating the expression of *β*-catenin. Moreover, PCA activated the cGMP pathway, thereby abrogating the detrimental impact of chronic stress on CRC (Cao et al., 2024). Despite these encouraging findings, more research is needed to confirm *L. johnsonii*’s preventive and treatment potential in CRC.

## 3 Inflammatory bowel disease

**Figure 2:**
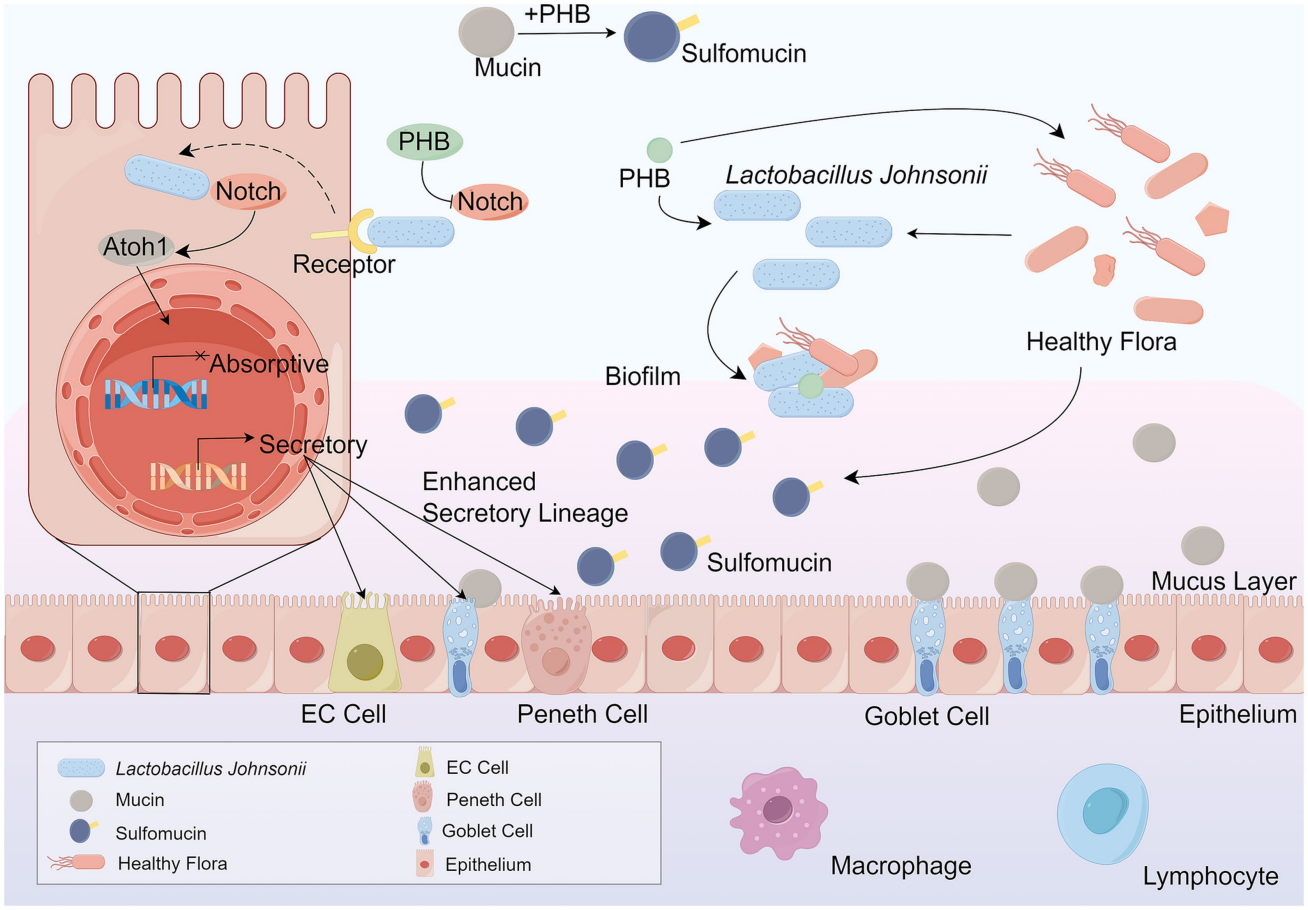
PHB mitigates diarrhea and colitis-associated inflammation by promoting the differentiation of goblet cells and enhancing sulfomucin production. This process is mediated by *L. johnsonii*, which utilizes PHB to establish a favorable ecological niche that supports biofilm formation and stabilizes its colonization in the intestinal mucosa. The biofilm formed by *L. johnsonii* enhances gut resilience by regulating fucose residue metabolism, thereby promoting goblet cell differentiation and sulfomucin maturation. Sulfomucins, as essential components of the mucus barrier, play a critical role in maintaining mucosal integrity and limiting pathogen invasion. Importantly, the anti-inflammatory effect of PHB is dependent on the presence of *L. johnsonii*, highlighting the synergistic relationship between microbial metabolites and beneficial bacteria in maintaining intestinal homeostasis. Through this pathway, PHB indirectly strengthens the mucosal immune barrier, attenuates colitis symptoms, and contributes to the host’s defense against gut inflammation. *L. johnsonii, Lactobacillus johnsonii*. PHB, poly-β-hydroxybutyrate. EC cell, enteroendocrine cell.

**Figure 3:**
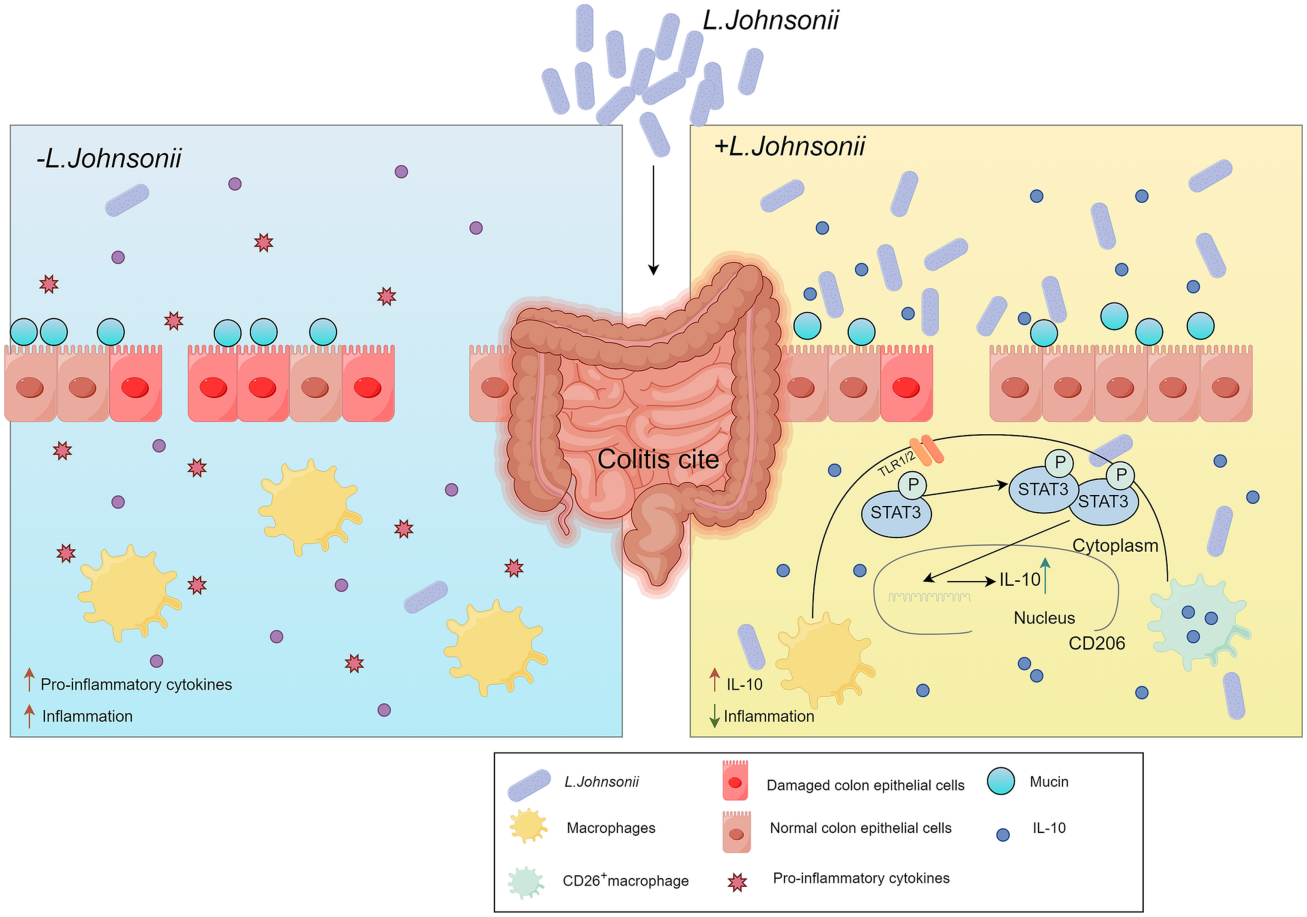
Schematic representation illustrating how resident *L. johnsonii* activates intestinal macrophages and promotes their polarization into CD206^+^ anti-inflammatory macrophages via the TLR1/2-STAT3 signaling pathway. This macrophage reprogramming enhances the secretion of IL-10, a key anti-inflammatory cytokine, thereby alleviating experimental colitis. *L. johnsonii*, *Lactobacillus johnsonii*. IL-10, interleukin-10.

Changes in the gut microbiota can arise from shifts in bacterial community diversity or microbiota-host interactions, and these changes are directly linked to various diseases, particularly IBD, which encompasses Ulcerative Colitis (UC) and Crohn’s Disease (CD) (Hoarau et al., 2016). Numerous hypotheses have been put forward regarding the effects of probiotics on IBD: (1) Probiotics modulate the intestinal immune system through regulating the expression of pro-inflammatory cytokines (Liu et al., 2011). (2) Probiotics regulate the microbial balance of the gastrointestinal tract by inhibiting the growth of pathogens (Chen et al., 2013). (3) Probiotics enhance the function of the intestinal barrier by increasing the expression of tight junction proteins (Bai et al., 2022). Additionally, glycoconjugates from the *Lactobacillus* cell wall act as antigens that may serve as novel diagnostic biomarkers for IBD (Paściak et al., 2017). Biosynthesized poly-*β*-hydroxybutyrate (PHB) alleviates inflammation by regulating fucose residues to promote goblet cell differentiation and sulfomucin maturation, providing a favorable environment for *L. johnsonii* biofilm formation as seen in Figure 2 (Ma et al., 2022). The supernatant of *L. johnsonii* culture alleviated colitis and remodeled gut microbiota, with increased SCFA production inhibiting the MAPK signaling pathway and M1 macrophage polarization (Wu et al., 2023). *L. johnsonii GLJ001* prevented dextran sulfate sodium (DSS)-induced colitis in mice by inhibiting M1 macrophage polarization via gut microbiota-SCFAs axis (Cai et al., 2025). Findings suggest that Butyrolactone-I (BTL-1) regulates intestinal flora, promotes the proliferation of *L. johnsonii*, safeguards the integrity of the intestinal barrier, increases the concentrations of butyric and propionic acids, and ultimately inhibits the activation of the MAPK signaling pathway in mice, thus alleviating IBD (Chen et al., 2025). In Muc2(−/−) mice, *L. johnsonii* demonstrates rapid growth, recovers to the initial population level and reduces the amount of *Enterococcus faecalis* (*E. faecalis*) (Makusheva et al., 2024). LJ-EVs can be directly internalized by intestinal epithelial cells and activate the Nrf2/HO-1 antioxidant signaling pathway, reducing endotoxin damage to cells and maintaining intestinal barrier homeostasis, which in turn alleviates the intestinal inflammation response (Li et al., 2024). There are fewer studies on LJ-EVs available. Future studies need to use sterile animal models or purified metabolite compartments to differentiate between the direct effects of *Lactobacillus* strains themselves and their metabolites or their potential synergistic effects. Additionally, research has revealed that *β*-Glucan ameliorates colitis via the *L. johnsonii*-indole-3-lactic acid (ILA)-aryl hydrocarbon receptor (AhR) axis (Zhang et al., 2024). Other studies have indicated that endoplasmic reticulum (ER) stress-related apoptosis is a crucial biological pathway in the pathophysiology of IBD (Cao et al., 2013; Ma et al., 2017). As shown in Figure 3. It has been demonstrated that *L. johnsonii* attenuated ER stress-related cell death (Zhang et al., 2021).

UC is a subtype of IBD characterized by persistent, nonspecific inflammation of the rectum and colon (Danese and Fiocchi, 2011; Ordás et al., 2012). Mounting evidence has revealed a significant correlation between *L. johnsonii* and the severity of UC (He et al., 2022). Research indicates that *L. johnsonii* activates resident macrophages, reprogramming them into CD206 + macrophages via the TLR1/2-the Signal transducer and activator of Transcription3 (STAT3) signaling pathway. This activation elicits the release of IL-10, effectively alleviating experimental colitis (Jia et al., 2022). The product ID-JPL934 comprises *L. johnsonii IDCC9203* (isolated from infant feces), which demonstrated the ability to the model for UC, including the constriction of the length of the colon, immune cell infiltration in the mucosa and submucosa, severe crypt damage, and loss of goblet and epithelial cells as observed in histological analysis (Je et al., 2018).

CD is another IBD that has seen a significant increase in incidence worldwide in recent years (Ng et al., 2017; Shouval and Rufo, 2017). Unlike UC, CD can affect the entire digestive tract, with transmural and segmental inflammation most commonly found in the terminal ileum and colon (Torres et al., 2017). Evidence suggests that the pathogenesis of CD is a result of gut inflammation caused by a combination of genetic and microbial factors, leading to abnormal immune regulation in the host (Ramos and Papadakis, 2019; Roda et al., 2020). Existing research suggests that the impact of *L. johnsonii* in the management of CD appears to be rather limited. The elemental diet (ED), widely utilized as an enteral nutritional intervention for CD, leads to a decrease in the population of *L. johnsonii*. This reduction is accompanied by a decline in the quantity and diversity of the intestinal microbiota, as well as a decrease in proinflammatory cytokines. Such changes are likely a consequence of alterations in the composition of lactic acid bacteria (Kajiura et al., 2009). A randomized, double-blind, placebo-controlled study, which spanned over 6 months, concluded that *L. johnsonii LA1* was not significantly effective, if at all, in preventing endoscopic recurrence of CD (Marteau et al., 2006). Another multicenter randomized-controlled clinical trial had the similar result, which failed to prevent early endoscopic recurrence 12 weeks after ileo-colorectal resection (Van Gossum et al., 2007). The reasons for the ineffectiveness of *L. johnsonii LA1* need to be further investigated. The potential influence of inter-individual differences in the patient’s gut microbiota may be a contributing factor.

The discrepancy between clinical and basic research findings may be influenced by multiple factors. First, strain-level variability should not be overlooked. Within the *L. johnsonii* species, substantial genomic diversity exists, leading to significant differences among strains in terms of metabolite production, colonization capacity, and immunomodulatory properties. Therefore, the anti-inflammatory effects demonstrated by certain strains in animal models or *in vitro* experiments may not be directly applicable to all clinical settings (Arzola-Martínez et al., 2024). Second, host-related factors also play a critical role. Individual differences among CD patients, including genetic background, gut microbiota composition, baseline inflammation levels, and postoperative recovery status, can affect the colonization efficiency and functional expression of *L. johnsonii*, thereby contributing to inconsistent intervention outcomes. Moreover, limitations in the design of existing clinical trials—such as short intervention durations, limited follow-up periods, and the lack of standardized dosages and delivery methods—may further obscure the potential long-term benefits of *L. johnsonii.*

In this context, future research should focus on precise strain selection, taking into account host-specific characteristics, and optimizing clinical trial protocols. Such efforts are essential to comprehensively evaluate the true therapeutic potential of *L. johnsonii* in CD management and to elucidate the fundamental reasons underlying the gap between mechanistic studies and therapeutic reality.

Psoriasis and IBD exhibit resemblances in terms of their commensal microbial communities and immune mechanisms. A prior investigation demonstrated that in the imiquimod (IMQ) mouse model (van der Fits et al., 2009; Van Belle et al., 2012; Terhorst et al., 2015), which is a murine model for psoriasis, the gut microbiome undergoes perturbations with reduced *L. johnsonii* and *Lactobacillus reuteri (L. reuteri)* populations (Kiyohara et al., 2019). This finding implies a potential connection between the skin microbiota and the gut microbiota in the context of psoriasis.

In summary, *L. johnsonii* exerts multifaceted regulatory effects on intestinal homeostasis in IBD, particularly by reshaping gut microbiota, strengthening barrier function, modulating immune responses, and alleviating inflammation through SCFA production and signaling pathway regulation. Despite promising results in UC models, its efficacy in Crohn’s disease and therapeutic reality s remains inconclusive. Future studies should prioritize clarifying strain-specific mechanisms, exploring microbial metabolite-host interactions, and investigating the role of *L. johnsonii* in extraintestinal diseases linked to gut dysbiosis, such as psoriasis.

## 4 *Helicobacter pylori* associated gastritis

*H. pylori* is a Gram-negative pathogen with spiral motility and ability to colonize acidic stomach. Chronic infection causes diseases, including chronic active gastritis, chronic atrophic gastritis, peptic ulcer and cancer. Previous studies have indicated that probiotics may hold potential in the treatment of *H. pylori* infection (Malfertheiner et al., 2002). *L. johnsonii* intake has a weak but favorable effect on associated gastritis (Pantoflickova et al., 2003), like *L. johnsonii La1* strain reducing colonization in children (Felley et al., 2001; Cruchet et al., 2003; Gotteland et al., 2008).

Regarding the mechanism of *L. johnsonii* inhibition of *H. pylori* associated gastritis, the current study did the following. As mentioned earlier, the surface GroEL protein of *L. johnsonii La1* has been identified to possess activities contributing to its probiotic characteristics. These activities include attachment to mucus and epithelial cells, stimulation of cytokine secretion from macrophages and epithelial cells, and the capacity to mediate the aggregation of the gastric pathogen *H. pylori*. *L. johnsonii La1* GroEL binds to mucins and intestinal HT29 cells in a pH-dependent manner: while a strong binding capacity was observed at pH 5.0, no binding occurred at pH 7.2. The recombinant GroEL (rGroEL) protein of *L. johnsonii La1 can* induce the aggregation of *H. pylori* (Bergonzelli et al., 2006). An acidic pH environment induces stress-sensitive GroEL expression and increases *L. johnsonii La1* death (Bergonzelli et al., 2006). In the early infection stages of *H. pylori*, *L. johnsonii La1* can reduce gastritis reducing macrophage inflammatory protein 2 (MIP-2) and keratinocyte-derived cytokine (KC) in serum and mucosa. Neutralized *L. johnsonii La1* spent culture supernatant reduces IL-8 secretion by human adenocarcinoma AGS cells, which may be through a reduction in pro-inflammatory chemotactic signals that recruit lymphocytes and neutrophils in the lamina propria (Sgouras et al., 2005). Both live and lyophilised strains of *L. johnsonii No. 1088 (HK-LJ88)* can inhibit *H. pylori* growth in mice, not through co-aggregation but an unknown mechanism involving cell surface molecules (Aiba et al., 2015; Aiba et al., 2017). Additionally, *HK-LJ88* can inhibit gastric acid secretion by reducing gastrin-positive cells (Aiba et al., 2015). The GroEL protein of *L. johnsonii La1* is a core effector for its gastric colonization and anti-Hp, which is distinct from other strains of Enterobacteriaceae such as *L531* and *BS15* in terms of molecular targeting. For example, LJ-EVs are important in activating the Nrf2/HO-1 antioxidant pathway induction, and M2 macrophage polarization. *L531* alters the composition of the intestinal microbiota and the production of SCFA, which attenuates intestinal infections. However, the specific adhesion mechanism with intestinal epithelial cells has not been clearly elucidated. *BS15*, on the other hand, possesses major functions in regulating metabolism and strengthening the intestinal mucosal barrier. Future studies could focus on their differences in molecular targets or mechanisms of action.

Studies also showed that *L. johnsonii 456* has higher viability at lower pH compared to other strains (Davoren et al., 2019), and proton pump inhibitors (PPIs) can reduce *L. johnsonii* and exacerbate indomethacin-induced small intestinal damage (Nadatani et al., 2019). Lactic acid from *L. johnsonii* plays a protective role in NSAID-induced small bowel injury. The discrepancy observed in these research outcomes can be ascribed to a diverse range of factors. For example, significant differences exist in assay results when examining the gut microbiota of mice versus humans. Additionally, variations in the bacterial homeostasis of the gastrointestinal mucosa among different individuals contribute to these discrepancies. These factors highlight the urgent need for further in-depth exploration, especially in identifying elements that foster the survival of gut microbiota and their adhesion to the gastrointestinal tract.

Based on the above, *L. johnsonii* exhibits strain-specific potential in mitigating *H. pylori*-induced gastritis by modulating host immune responses, interfering with pathogen adhesion, and influencing gastric environment via factors like GroEL protein and lactic acid production. While strains such as *L. johnsonii La1* and *No. 1088* have shown efficacy in reducing *H. pylori* colonization and associated inflammation, variations in outcomes across studies underscore the complexity of host–microbiota–pathogen interactions. Future research should focus on elucidating the molecular pathways underpinning these probiotic effects, optimizing strain viability under gastric conditions, and identifying host-specific factors influencing probiotic colonization and therapeutic efficacy. Personalized approaches integrating microbial and host biomarkers may ultimately improve probiotic strategies against *H. pylori*.

## 5 Liver disease

### 5.1 Alcoholic liver disease

Excessive alcohol consumption has long been recognized as a firmly established risk factor for the development of chronic liver disease (Cojocariu et al., 2014). Oxidative stress and lipid peroxidation are pivotal pathogenic mechanisms underpinning the formation of fatty liver (Wu and Cederbaum, 2003; Albano, 2006). Malondialdehyde (MDA) is a biomarker for oxidative stress. A study found that heat-killed *L. johnsonii* is capable of countering the elevation of alanine transaminase (ALT), aspartate transaminase (AST), gamma - glutamyl transferase (*γ*-GT), MDA, and triglyceride (TG) within the HepG2 cell line, which represents a human hepatocellular carcinoma cell line. In animal experiments, *L. johnsonii* effectively reduced γ-GT levels but had no significant impact on AST and ALT (Chuang et al., 2016). Heat-killed *L. johnsonii* upregulated intestinal lysozyme expression. This enhanced the production of immunoregulatory substances by gut bacteria, activating the Nucleotide-binding oligomerization domain 2 (NOD2)-interleukin (IL)-23-IL-22 innate immune axis. Elevated IL-22 increased antimicrobial peptide synthesis for intestinal homeostasis and activated STAT3 pathway in the liver, promoting hepatic injury repair (Yin et al., 2025).

### 5.2 Metabolic dysfunction-associated fatty liver disease

Non-alcoholic fatty liver disease (NAFLD), now re-designated as metabolic dysfunction-associated fatty liver disease (MAFLD) (Eslam et al., 2020), is a liver disorder of global prevalence that exhibited a robust association with obesity (Lazo et al., 2013). *L. johnsonii BS15 (BS15)*, isolated from yogurt, has manifested beneficial effects in murine models. It can bring about a reduction in body weight, liver weight, and fat content. In hyperlipidemic mice, *BS15* safeguards against hepatic steatosis and apoptosis. In MAFLD mice, *BS15* mitigates mitochondrial abnormalities by diminishing the levels of uncoupling protein-2 and cytochrome c. Additionally, *BS15* modulates intestinal permeability and the composition of the gut microbiota, thereby resulting in a decline in serum lipopolysaccharide and inflammatory factors (Xin et al., 2014). Another research investigation indicates that the combination of *BS15* and abdominal massage treatment in MAFLD model rats can decrease cholesterol levels and liver injury indices, enhance intestinal permeability, fortify the intestinal mucosal barrier, and facilitate the repair of hepatocyte biofilms (Zhang et al., 2021).

Given the above, *L. johnsonii* exhibits promising hepatoprotective effects in both alcoholic and MAFLD. In alcoholic liver injury, heat-killed *L. johnsonii* can alleviate oxidative stress and modulate immune responses through the NOD2–IL-23–IL-22 axis, contributing to intestinal homeostasis and hepatic repair. In MAFLD models, the *BS15* strain demonstrates metabolic benefits by reducing lipid accumulation, improving mitochondrial function, and strengthening the gut barrier. These findings underscore the strain-specific and multi-targeted potential of *L. johnsonii* in liver disease management. Future research should focus on clinical validation and exploring personalized probiotic interventions that account for host metabolic and microbial variability.

Notably, MAFLD often coexists with diabetes mellitus, and both share the core pathologic basis of insulin resistance and intestinal dysbiosis. Although the present study primarily focused on the role of *L. johnsonii* in a simple MAFLD model, its demonstrated ability to improve insulin sensitivity, regulate lipid metabolism, and repair the intestinal barrier and flora suggests its potential value as an adjunctive means to intervene in diabetes mellitus complicating MAFLD. However, the assessment of specific effects and microbiota regulatory capacity in this complex co-morbid state is uniquely challenging and requires subsequent specialized studies.

## 6 Obesity and lipid metabolism

Obesity and related metabolic disorders result from excessive caloric intake and insufficient physical activity, leading to fat accumulation and various diseases (Xu et al., 2003). Evidence shows probiotics, including *Lactobacillus*, help restore metabolic and immune function (Dahiya et al., 2017; Park et al., 2018, 2019). *L. johnsonii*, from human and animal intestines, has strains with probiotic properties (Fukushima et al., 2007; Buhnik-Rosenblau et al., 2012; Fonseca et al., 2017; Zheng et al., 2020). Its progress in obesity metabolism and fatty liver disease is as follows.

*Lactobacillus johnsonii* has anti-obesity and cholesterol-lowering effects. *L. johnsonii JNU3402 (LJ3402)*, from Korean infant feces, is bile and acid resistant. In high-fat diet mice, non-viable *LJ3402* was observed to reduce liver weight, as well as epididymal white adipose tissue (WAT), regulate triglyceride levels, and slightly improve insulin resistance. The anti - obesity effect of LJ3402 is mediated via the PPARγ pathway. This pathway not only enhances mitochondrial function within WAT but also promotes thermogenesis (Yang et al., 2020). Phycobiliproteins (derived from arthrospira platensis) bioactive peptide extracts (PPE) can increase *L. johnsonii* abundance and is a potential supplement for obesity (Liu et al., 2022). Another study linked hypercholesterolemia to liver cholesterol metabolism. *L. johnsonii BFE6154* exerts its cholesterol - lowering effect by downregulating the expression of the Niemann-Pick C1-like 1 (NPC1L1) gene. It achieves this by activating the liver X receptor (LXR) in liver cells, thereby reducing cholesterol uptake. Furthermore, the metabolite of *L. johnsonii BFE6154* stimulates the expression of the low density lipoprotein receptor (LDLR) gene in hepatocytes, facilitating enhanced cholesterol clearance. In the intestine, *L. johnsonii BFE6154* upregulates the ABCG5 gene while downregulating the ABCG8 and NPC1L1 genes, effectively curbing intestinal cholesterol absorption. These findings have substantially enhanced our understanding of the complex cholesterol-lowering mechanisms of *L. johnsonii* (Yoon et al., 2023).

The high degree of co-morbidity between diabetes mellitus and MAFLD and their common pathophysiologic basis (insulin resistance, chronic low-grade inflammation, intestinal flora dysbiosis, intestinal barrier damage, and gut-hepatic axis disturbances). This overlapping of multiple pathologies creates more complex stresses on host metabolism and the intestinal microenvironment, challenging traditional monotherapies. *L. johnsonii* has shown potential in modulating obesity and related metabolic disorders such as insulin resistance, dyslipidemia (e.g., strain *JNU3402*, *BFE6154*). These metabolic benefits, together with its potential hepatoprotective effects, make it one of the candidate probiotics for exploring interventions in the obesity-diabetes-fatty liver “metabolic co-morbidity syndrome.” Strains like *L. johnsonii* with potential to modulate metabolism and the gut environment serve as potential adjunctive intervention strategies. Future studies need to deeply explore their effects and mechanisms in the superimposed state of multiple metabolic disorders.

In a word, *L. johnsonii* exhibits notable potential in combating obesity and related metabolic disorders through multiple mechanisms. Specific strains such as *LJ3402* demonstrate anti-obesity effects by enhancing mitochondrial function and promoting thermogenesis via the PPAR*γ* pathway, while *BFE6154* exerts cholesterol-lowering activity by modulating key hepatic and intestinal genes involved in lipid absorption and metabolism. Additionally, dietary interventions like phycobiliprotein-derived peptides may further augment *L. johnsonii* abundance, offering a synergistic approach to metabolic regulation. These findings highlight the multifaceted metabolic benefits of *L. johnsonii*, warranting further investigation into its clinical efficacy and application in personalized metabolic therapies.

## 7 Other organs

### 7.1 Brain-gut axis

The gut microbiota is crucial for regulating brain function, especially in emotional processing and behavior (Dinan and Cryan, 2012; Steenbergen et al., 2015; Bharwani et al., 2016). There is growing evidence for a “microbe-gut-brain axis, “where gut microbiota influence central nervous system function by modulating neuroimmune function, neuronal signaling, and metabolic activity (Fang et al., 2020). The relationship between gut microbiota, including *L. johnsonii*, and brain function offers new approaches and potential for gut microbiota-based therapies for brain-related diseases.

**Figure 4:**
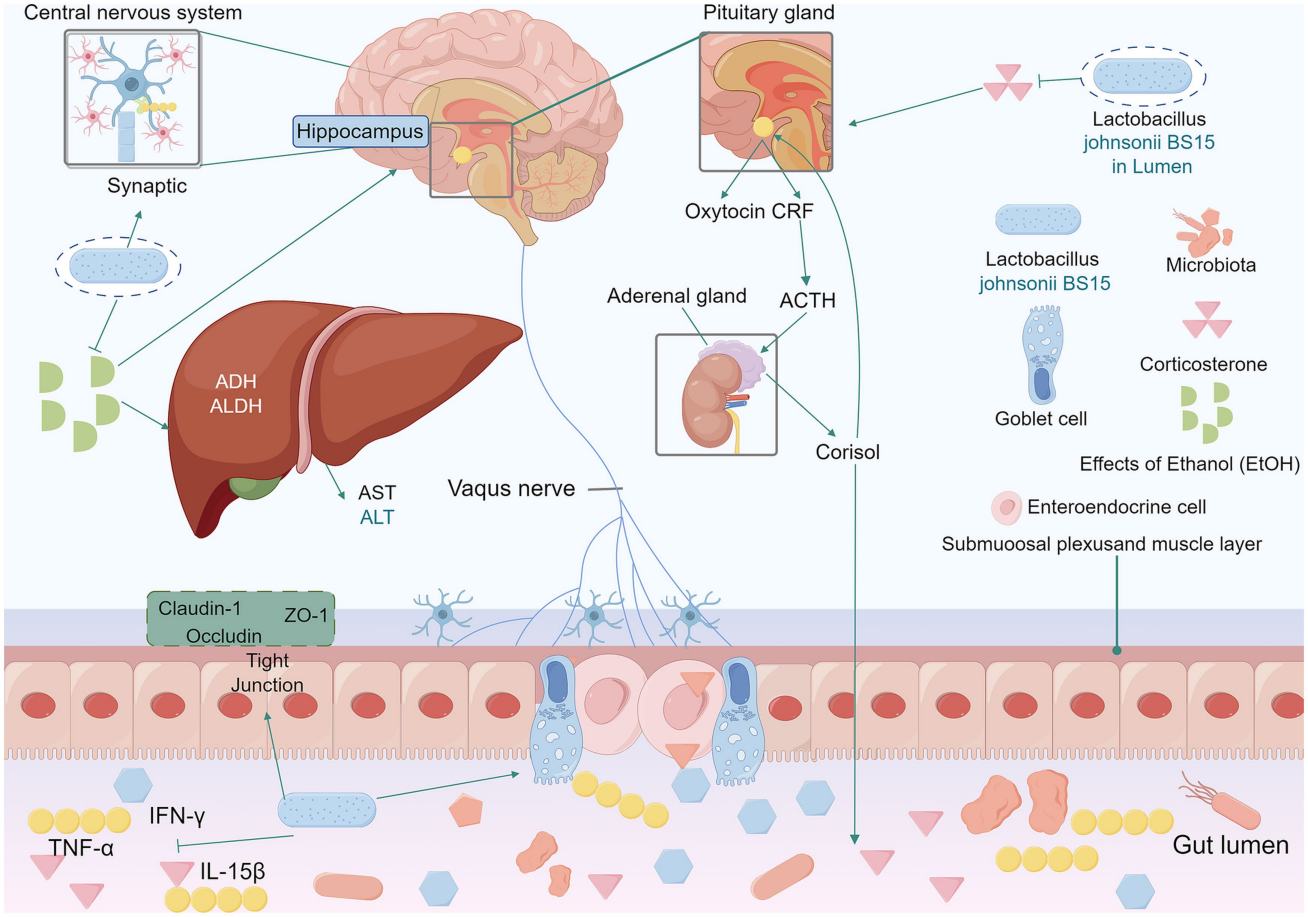
In mice exposed to high levels of fluoride, *L. johnsonii BS15* increased the number of small intestinal cuprocytes and constituted an effective intestinal barrier against fluoride-induced intestinal injury. *BS15* positively regulated the hypothalamo-pituitary–adrenal axis by lowering the serum corticosterone level. *BS15* also reduced the levels of TNF-*α*, IFN-*γ* and IL-15β in the mouse small intestine. Enhanced gut development, modulat mRNA expression levels of the tight junction proteins claudin-1, occludin, and ZO-1. Lower gut microbiota, and blood LPS levelsIn the intestine, BS15 prevents intestinal barrier damage in mice by increasing mRNA levels of tight junction proteins and exerts a beneficial effect on reducing pro-inflammatory cytokine levels. *BS15* improves the duration of ethanol resistance and the activities of ADH and aldehyde dehydrogenase ALDH in the liver of acute alcohol-induced mice. Increases ethanol metabolism levels to ameliorate alcohol-mediated memory impairment. *L. johnsonii BS15, Lactobacillus johnsonii BS15*. ADH, alcohol dehydrogenase. EtOH, effects of ethanol. AST, aspartate aminotransferase. ALT, alanine aminotransferase. ADH, alcohol dehydrogenase. ALDH, aldehyde dehydrogenase. TNF-*α*, tumor necrosis factor-*α*. IFN-*γ*, interferon gamma. IL-15*β*, Interleukin-15*β*.

Psychological stress can impair cognitive function (Sandi and Pinelo-Nava, 2007; Mo et al., 2013; Guo et al., 2017). A study shows *BS15* can address stress-induced memory disorders via the brain-gut axis. *BS15* pretreated group had increased trypsin and lipase activity, more cupped cells, and improved jejunum and ileum development. *BS15* further led to a reduction in cytokine levels within the small intestine and regulated the expression of memory-related functional proteins. *BS15* also restored antioxidant capacity and protected against mitochondria-mediated apoptosis in the hippocampus (Wang et al., 2020). It enhanced memory test performance under stress and regulated the hypothalamic–pituitary–adrenal axis (Wang et al., 2021). Excessive fluoride intake can cause chronic fluorosis and neurotoxicity (Queste et al., 2001). In mice exposed to high levels of fluoride, *BS15* increased the number of goblet cells, decreased the secretion of sIgA, and protected against fluoride-induced intestinal damage and memory deficits (Sun et al., 2020). Chronic alcohol consumption disrupts intestinal microbes and impairs brain memory (Stärkel and Schnabl, 2016; Neyrinck et al., 2017; Scholey et al., 2019). *BS15* increased EtOH metabolism, shortened the duration of alcohol-induced coma and blood alcohol concentration, and alleviated memory impairment (Sun et al., 2022). As illustrated in Figure 4. Furthermore, *BS15* attenuated 2,4,6-trinitrobenzenesulfonic acid (TNBS) and *E. coli*-induced memory impairment and colitis and restored gut microbiota balance. Treatment with *L. johnsonii* reduced LPS levels and restored disrupted gut microbiota (Jang et al., 2018). Taken together, these findings strongly suggest that *BS15* has the potential to ameliorate stress-related memory disorders and holds great promise for the treatment of diseases associated with memory impairment.

There are studies on the specific serotonergic pathways within the brain-gut axis, vagus nerve-mediated mechanisms, etc. still lacking. Future studies should focus on finding more direct evidence of molecular interactions, such as changes in receptor expression or neurotransmitter levels.

### 7.2 Heart and lung

In addition to the brain-gut axis, *L. johnsonii* also plays a role in regulating the functions of other organs. The “gut-heart axis” is a novel concept that provides insights into the complex mechanisms of acute myocardial infarction (AMI) (Zununi Vahed et al., 2018). The intestinal barrier is a pivotal component in the communication network between the gut microbiota and the heart (Lewis and Taylor, 2020). A specific study has shown that a 28-day intervention with *L. johnsonii* effectively preserved cardiac function, retarded the development of cardiac pathological changes, suppressed the production of cytokines associated with myocardial injury, and enhanced the integrity of the intestinal barrier. Notably, this treatment also reshaped the gut microbiota, leading to a significant increase in the relative abundance of *L. johnsonii* (Zhong et al., 2023).

Accumulating experimental and epidemiological data highlight a strong connection between the gut microbiota and the lungs, which is commonly referred to as the “gut-lung axis” (Dang and Marsland, 2019). Published research has demonstrated that intranasal administration of *L. johnsonii* can confer protection against hyperoxia - induced lung injury and modulate the composition of the gut microbiota, suggesting a potential therapeutic strategy for lung-related disorders (Chen et al., 2023).

To sum up, *L. johnsonii* exerts beneficial effects beyond the gut, notably through the brain-gut, gut-heart, and gut-lung axes. Strain *BS15* has demonstrated the ability to alleviate stress-induced memory impairment, counteract neurotoxicity from fluoride and alcohol exposure, and restore cognitive function by modulating the hypothalamic–pituitary–adrenal axis, enhancing insstestinal barrier integrity, and improving antioxidant defenses in the hippocampus. Additionally, *L. johnsonii* supports cardiovascular health by reinforcing the gut barrier, reducing myocardial inflammation, and preserving cardiac function post-AMI. In respiratory contexts, intranasal *L. johnsonii* administration mitigates hyperoxia-induced lung injury while positively shaping gut microbial composition. Collectively, these findings underscore the systemic regulatory capacity of *L. johnsonii*, suggesting promising therapeutic applications in neuroprotection, cardioprotection, and pulmonary health via microbiota-mediated pathways.

## 8 Summary and outlook

*Lactobacillus johnsonii* plays a critical role in gastrointestinal (GI) health through immunomodulatory mechanisms, reinforcement of intestinal barrier functionality, and attenuation of inflammatory responses. Although the specific effects vary depending on the strain and application context, this review reveals that the potential common mechanisms underlying the probiotic functions of *L. johnsonii* primarily include: regulating the composition and function of the gut microbiota (e.g., increasing beneficial bacteria and inhibiting pathogenic bacteria); enhancing or repairing the intestinal barrier (e.g., promoting the expression of tight junction proteins and increasing mucus secretion); modulating host immune responses (e.g., regulating cytokine secretion and influencing immune cell differentiation); producing bioactive metabolites (SCFAs, ILA, PCA, etc.); and mediating distant organ effects. However, translating preclinical and clinical findings into robust applications faces significant hurdles, including strain-specific variability in probiotic efficacy (e.g., divergent outcomes between *L. johnsonii La1* and *BS15* in IBD management), inconsistent clinical trial results (such as the failure of *L. johnsonii La1* to prevent CD recurrence), and fragmented regulatory frameworks for validating health claims. These challenges underscore the need for strain-specific characterization, precised strain identification and functional validation, personalized probiotic strategies aligned with individual microbiota profiles, and harmonized safety and efficacy standards across jurisdictions. In summary, despite significant differences in the actions of different strains of *L. johnsonii*, the available research strongly suggests that the strain as a whole holds core effects on great potential to improve digestive health and associated systemic diseases through the multiple mechanistic modules mentioned above (flora modulation, barrier repair, immunomodulation, metabolite production, other organs) Potential. The positive effects (e.g., anti-infective, anti-inflammatory, metabolic regulation, neuroprotection) demonstrated by many strains in specific models or clinical situations provide an important basis and direction for further research and development of this strain.

Despite established anti-inflammatory mechanisms mediated through TLR/STAT3 signaling and ER stress modulation, critical gaps persist in elucidating the bioactive metabolites (e.g., propionic acid, bacteriocins) and host–microbe interactions underlying *L. johnsonii*’s effects. Current research is further constrained by overreliance on animal models and small-scale human trials, which may fail to recapitulate human pathophysiology or account for microbiome heterogeneity. Further, the potential impact of host genetic factors (e.g., polymorphisms in NOD2, TLR4) on strain efficacy remains worth exploring. In case the results of the study may be biased. Additionally, the interplay between *L. johnsonii* and comorbid conditions, such as metabolic disorders or mental health issues, remains underexplored, necessitating advanced multi-omics approaches to identify predictive biomarkers.

*Lactobacillus johnsonii* also has an interaction network with other major symbiotic bacteria, such as *Bifidobacterium* or *Bifidobacterium*. Charlet et al. (2020) found that *L. johnsonii* can co-secrete chitinase and mannosidase with *Bacteroides thetaiotaomicron* to degrade fungal cell walls. This significantly inhibits the overgrowth of Candida species and reduces the abundance of pathogenic bacteria such as *E. coli* and *Enterococcus*, thereby alleviating colitis symptoms in mice (Charlet et al., 2020). In addition to its local effects, *L. johnsonii* can also influence systemic diseases through microbiota remodeling. For example, in a rat model of AMI, *L. johnsonii* can increase the abundance of *Muribaculaceae* and *Lactobacillus* while decreasing the levels of potential pathogens such as *Romboutsia* and *Clostridia*, thereby improving cardiac function and demonstrating its potential to modulate the function of distal organs through the “gut-heart axis” (Zhong et al., 2023). In terms of competitive colonization, *L. johnsonii* can bind tightly to host epithelial cells via surface proteins, which may affect the ecological niches occupied by other microorganisms. The surface “moonlighting protein” GAPDH of *L. johnsonii* can bind to the tight junction protein JAM-2 of intestinal epithelial cells, thereby enhancing intestinal barrier function and providing an advantage for its stable colonization (Bai et al., 2022). In addition, this strain exhibits potent antimicrobial capabilities, inhibiting a variety of pathogenic microorganisms, including *H.pylori*, *E. coli*, *Salmonella*, and *Clostridium difficile*, thereby further altering the gut microbiota structure (Aiba et al., 2015).

Future research priorities include large-scale, double-blind randomized controlled trials (RCTs) to evaluate strain- and disease-specific efficacy in IBD subtypes, *H. pylori* eradication, and metabolic syndrome. Meanwhile, future therapeutic reality of *L. johnsonii* necessitate meticulous strain selection, as distinct strains (e.g., *L. johnsonii La1, L531, BS15*) exhibit divergent therapeutic effects across digestive diseases, underscoring the importance of strain-specific mechanistic studies and rigorous clinical validation to optimize probiotic efficacy and safety. Mechanistic studies should integrate genomics, proteomics, and metabolomics to map strain-specific pathways, such as GroEL-mediated pathogen aggregation or GAPDH-JAM-2 interactions in epithelial barrier repair. Concurrent efforts to optimize industrial-scale production through culture condition refinement or genetic engineering could enhance probiotic viability and functionality. Safety assessments in vulnerable populations (e.g., immunocompromised individuals, neonates) are imperative to address risks like horizontal gene transfer or opportunistic infections. Finally, interdisciplinary collaboration is essential to develop standardized guidelines for probiotic use, validate functional food formulations, and establish regulatory pathways that balance innovation with public health safeguards. By addressing these challenges, *L. johnsonii* may transition from a promising probiotic to a cornerstone of precision medicine in gastrointestinal and systemic health management.

In conclusion, *L. johnsonii* emerges as a clinically significant probiotic strain that demonstrates critical efficacy in human health maintenance, particularly in the context of gastrointestinal disorder management, while exhibiting substantial translational potential for therapeutic applications in digestive tract pathologies.

Further research should prioritize elucidating strain-dependent molecular pathways and conducting large-scale trials to establish tailored therapeutic strategies for targeted disease interventions.

## Glossary

5-FU: 5-fluorouracil
AhR: aryl hydrocarbon receptor
ALT: alanine transaminase
AMI: acute myocardial infarction
AST: aspartate transaminase
*BS15*: *L. johnsonii BS15*
BSH: Bile-Salt-Hydrolase
BTL-1: Butyrolactone-I
*C. jejuni*: *Campylobacter jejuni*
*C. rodentium*: *Citrobacter rodentium*
CD: Crohn’s Disease
CRC: colorectal cancer
DSS: dextran sulfate sodium
*E. coli*: *Escherichia coli*
*E. faecalis*: *Enterococcus faecalis*
EF-Tu: elongation factor Tu
ED: elemental diet
EHEC: enterohaemorrhagic *Escherichia coli*
EIEC: enteroinvasive *Escherichia coli*
ER: endoplasmic reticulum
GAPDH: glycerol-3-phosphate dehydrogenase
GI: gastrointestinal
*H. pylori*: *Helicobacter pylori*
*HK-LJ88*: *L. johnsonii No. 1088*
IBD: inflammatory bowel disease
IECs: intestinal epithelial cells
IL: interleukin
ILA: indole-3-lactic acid
IMQ: imiquimod
KC: keratinocyte-derived cytokine
*L. acidophilus*: *Lactobacillus acidophilus*
*L. johnsonii*: *Lactobacillus johnsonii*
*L. reuteri*: *Lactobacillus reuteri*
LDLR: low density lipoprotein receptor
*LJ3402*: *L. johnsonii JNU3402*
LJ-EVs: *L. johnsonii* derived extracellular vesicles
LPS: lipopolysaccharide
LTAs: lipoteichoic acids
LXR: liver X receptor
MAFLD: metabolic dysfunction-associated fatty liver disease
MAPK: Mitogen-Activated Protein Kinase
MDA: Malondialdehyde
MIP-2: macrophage inflammatory protein 2
NAFLD: Non-alcoholic fatty liver disease
NEC: Necrotizing enterocolitis
NOD2: Nucleotide-binding oligomerization domain 2
NPC1L1: Niemann-Pick C1-like 1
PCA: protocatechuic acid
PHB: poly-β-hydroxybutyrate
PPARγ: peroxisome-activated receptor-γ
PPE: peptide extracts
PPIs: proton pump inhibitors
RCTs: randomized controlled trials
rGroEL: recombinant GroEL
*S. typhimurium*: *Salmonella Typhimurium*
SBS: short bowel syndrome
sCD14: soluble CD14
SCFAs: short-chain fatty acids
STAT3: the Signal transducer and activator of Transcription3
TG: triglyceride
TLR3: Toll-like receptors 3
TNBS: 2,4,6-trinitrobenzenesulfonic acid
Treg: regulatory T
UC: Ulcerative Colitis
WAT: white adipose tissue
γ-GT: gamma - glutamyl transferase
